# Submillisievert CT chest for COVID-19 patients in a rural hospital with limited resources

**DOI:** 10.1186/s43055-022-00737-9

**Published:** 2022-03-04

**Authors:** Sammy Tawk, Wissam Mansour, Dayana Sleiman, Setrida Gemayel, Edgard Lozom, Karl El Mendelek, Nicole Saliba, Charbel Mourad

**Affiliations:** 1 Radiology Department, Université Catholique de Louvain, Namur, Belgium; 2 Division of Pulmonary, Allergy, and Critical Care Medicine, Duke University School of Medicine, Durham, NC USA; 3 Radiology Department, Bshary Governmental Hospital, Bshary, Lebanon; 4 Emergency Medicine Department, Bshary Governmental Hospital, Bshary, Lebanon; 5 Administration, Bshary Governmental Hospital, Bshary, Lebanon; 6 Faculty of Medical Sciences, Lebanese University, Beirut, Lebanon

**Keywords:** COVID-19, Chest CT, Ultra-low dose CT scan, As low as reasonably achievable (ALARA), Submillisievert

## Abstract

**Background:**

This is a secondary analysis of prospectively acquired data approved by the hospital institutional board committee. We performed a retrospective chart review of 463 patients who underwent a CT Chest for suspected COVID-19 infection between April 1st, 2020, and March 31st, 2021. Patients were grouped based on the CT chest obtained protocol: ultra-low dose or full dose. The likelihood of suspicion of COVID-19 infection was classified on a Likert scale based on the probability of pulmonary involvement. For each group, the sensitivity and specificity of CT were compared to nasopharyngeal swab as standard of reference. The median dose length product and duration of apnea were compared between both groups using two-tailed Mann–Whitney *U* test. The aim of this study is to share our experience of reducing radiation dose in COVID-19 patients by using an ultra-low dose CT chest protocol on a 16 row multidetector CT scan in a hospital with limited resources.

**Results:**

Two hundred sixty-nine patients underwent a full dose CT and 194 patients an ultra-low dose CT. In the former group, the median dose length product was 341.11 mGy*cm [Interquartile range (IQR), 239.1–443.2] and the median duration of apnea was 13.29 s [IQR, 10.85–15.73]. In the latter group, the median dose length product was 30.8 mGy*cm [IQR, 28.9–32.7] and median duration of apnea was 8.27 s [IQR, 7.69–8.85]. The sensitivity of the ultra-low dose CT was 91.2% and that of the full dose was 94%.

**Conclusion:**

A 90% reduction in estimated dose and 38% reduction in apnea duration could be achieved using an ultra-low dose CT chest protocol on a 16-row MDCT without significant loss in the sensitivity of CT to detect COVID-related parenchymal involvement.

## Introduction

In December 2019, the new coronavirus strain (SARS-Cov-2) was first reported in Wuhan, China [1]. The virus has spread rapidly across continents and was declared a pandemic by the World Health Organization (WHO) on March 12, 2020 [2]. In an effort to mitigate the spread of SARS-COV-2, the medical community has relied heavily on early viral detection and patient isolation. Biological testing using reverse transcriptase polymerase chain reaction (RT-PCR) has been the standard method of reference for diagnosis. The accuracy and predictive value of these tests may vary depending on the clinical setting, and specimen site [3]. They appear to be highly specific but may have a false negative rate in 10–40% of patients with COVID-19 [4].

Similar to many areas of the world, the laboratory of Bshary Governmental Hospital lacked RT-PCR facilities, and the performed nasopharyngeal swab consisted of limited number of specimens that were weekly sent to a central laboratory with delays in results [5]. This delay hindered a timely management and patient’s triage. CT scan emerged as a useful tool for early diagnosis of COVID-19 as reported in papers published soon after the start of the pandemic [6–8]. Unenhanced chest CT was performed on a Neusoft Classic CT scan with standard protocol detailed in Table 1. Clinicians relied on chest CT in addition to RT-PCR and rapid antigen tests in the triage and management of patients as reported by several institutions [7–18]. Following data from pilot studies demonstrating the high accuracy of ultra-low dose CT (LDCT) in COVID-19 diagnosis, and in application of the As Low As Reasonably Achievable (ALARA) principle [6, 8, 10, 13, 19, 20], a LDCT chest protocol was implemented as of December 2020 to achieve an estimated dose inferior to 1 millisievert.

This paper aims to share our experience of reducing radiation dose in COVID-19 patients by using an ultra-low dose CT chest protocol on a 16 row multidetector CT scan in a hospital with limited resources.

**Table 1:** Acquisition parameters on 16-row multidetector CT scan (Neuviz-Classic) for the regular full dose protocol (FDCT) and the modified ultra-low dose protocol (LDCT)

	FDCT	LDCT
Tube kilovoltage (Kv)	120	80
Current (mAs)	Automatic current modulation	Fixed: 15–50
Pitch	1	1.5
Rotation speed	0.78 s	0.78 s
Scan coverage	Full coverage of lung fields: neck base to upper abdomen	Strict coverage of lung fields. Skipping of lung apices or cul-de-sac is tolerated
Image reconstruction (Kernel)	F20 (3 mm) and F70 (1.25 mm)	F20 (3 mm) and F70 (3 mm)
Direction	Craniocaudal	caudocranial

FDCT, full dose CT scan; LDCT, ultra-low dose CT scan, estimated effective dose < 1 mSv

## Materials and methods

This is a single center secondary retrospective analysis of prospectively acquired data approved by the hospital institutional board committee in Bshary Governmental Hospital, located in a mountainous area in the district of Bshary in North Lebanon.

### Population

From April 1st, 2020, to March 31st, 2021, all patients with clinical suspicion for COVID-19 infection who underwent a CT scan of the chest in the radiology department at our institution (*N* = 463) were included in this study. Indication for imaging included an initial evaluation for suspected COVID-19 infection or evaluation for pulmonary involvement. Nasopharyngeal swabs for COVID-19 performed within 7 days of the CT, either before or after, was available for 168 patients (36.3%). RT-PCR was obtained for 54 patients and Rapid Antigen test (Roche) for 114 patients with a mean duration of 2.17 days ± 2.25 SD between CT and biological testing.

The duration of symptoms was clearly documented for 63 patients (13.6%) with a median of 5 days [IQR: 3–7] from symptom onset to CT.

### CT protocol

The radiology department at Bshary Governmental Hospital is equipped with a Neusoft Classic 16-detector CT scan (NeuViz, China). The regular full dose CT (FDCT) chest protocol is performed with the following parameters: 120 Kilovolts (KV), automatic current modulation (O-Dose), pitch of 1 and a scan coverage area from above the lung apices to below the pleural cul-de sac. The scan duration average is ~ 15 s. Image reconstruction is carried out using an iterative reconstruction algorithm provided by the manufacturer (ClearView). Two sets of images are obtained: 1.25 mm contiguous images in a sharp kernel (Lung) and 3 mm contiguous images in a smooth kernel (Mediastinum).

The ultra-low dose protocol was performed using the following parameters: tube kilovoltage: 80 kV, a fixed tube current ranging between 15 and 50 Milliampere-seconds (mAs) according to body habitus, as estimated by the technologist. Pitch of 1.5 and rotation speed of 0.78 s were used. The scan coverage area was limited to lung fields. Image reconstruction was carried out using an iterative reconstruction algorithm provided by the manufacturer (ClearView). Two sets of images were obtained: 3 mm contiguous images in a sharp kernel (Lung) and 3 mm contiguous images in a smooth kernel (Mediastinum). The 3 mm image thickness was used instead of 1.5 mm in order to compensate for the increase in image noise related to the low dose protocol. This compromise in image quality is accepted in the setting of the COVID-19 pandemic, since the analysis is focused on the lung parenchyma at the expense of a decreased image quality of the mediastinum, upper abdomen, and bones. In addition, the pitch has been increased while the scan coverage has been decreased and performed in a caudo-cranial acquisition. These modifications decreased the acquisition time and therefore the respiratory artifacts. The trade-off is a decrease in lung interstitial visibility and micronodules conspicuity.

### Image analysis

**Fig. 1:**
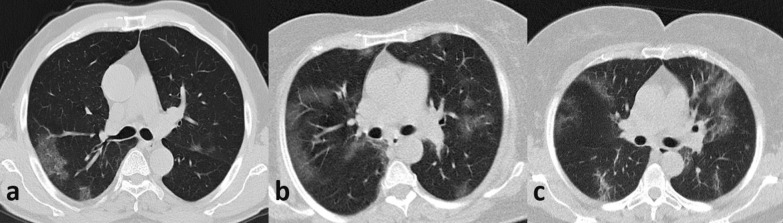
Example cases of three patients with typical findings of COVID-19 in FDCT (**a**) and LDCT (**b**, **c**). Adequate image quality is obtained both in full-dose and ultra-low dose CT chest protocols. **a** FDCT—normal BMI—DLP: 192 mGy*cm. **b** LDCT—normal BMI—DLP: 20 mGy*cm. **c** LDCT—Elevated BMI—DLP: 26 mGy*cm

CT images were prospectively interpreted by one of two radiologists with 2 and 5 years of experience, taking into consideration the available clinical information and the result of the nasopharyngeal swab if available at the time of CT interpretation (*N* = 71). Signs of COVID-19 infection included ground glass opacities in a multifocal peripheral distribution, crazy paving, vascular dilation, subpleural bands and consolidations (Fig. 1) [7, 21–30]. A semi-quantitative evaluation of parenchymal involvement was also performed. A categorical assessment scheme was used to conclude the study regarding the probability of COVID-19 infection as follows: 1: normal exam, 2: other infection is more likely, 3: indeterminate for COVID-19, moderate probability, 4: highly suggestive of COVID-19, 5: typical findings for COVID. In this paper, scores ≥ 3 were considered positive for COVID-19 [31].

### Data acquisition

Investigators who were blinded to the patient’s group (full or low dose CT) reviewed all medical charts, laboratory data, and biological test results of the patients. We also extracted chest CT dose summary sheets, scanning protocols, and the acquisition parameters: KV, mAs, Pitch, Rotation speed, breath hold duration, DLP and volume CT dose index (CTDI_vol_). The estimated effective dose was calculated by multiplying the DLP by a conversion factor (*K* = 0.014 for the chest) [32].

### Data analysis

A threshold of 1 millisievert was used to divide our population in two groups. There were 269 regular CT exams with a full dose (FDCT; estimated dose ≥ 1 millisievert) and 194 exams with an ultra-low dose CT (LDCT; estimated dose < 1 millisievert). The biological test (RT-PCR or Rapid Antigen test) was considered our reference standard for the diagnosis of COVID-19 infection.

### Statistical analysis

Descriptive analysis was described as continuous variables expressed as median (25th-75th percentile) and categorical variables represented as number (percentage) of participants. Normality of the distribution was assessed using the Shapiro–Wilk test. Mann–Whitney *U* test and Fisher Exact test were used to compare the median and percentages, respectively. A *P* value < 0.05 was considered statistically significant.

## Results

### Population characteristics

The demographic data of the FDCT group (*N* = 269) and the LDCT group (*N* = 194) are summarized in Table 2. Biological tests were positive in 130/168 (77.4%) patients. CT scan was positive (score ≥ 3) in 305/463 patients (65.9%). Using a semi-quantitative visual evaluation, 34% had < 10% parenchymal involvement, 30% had 11–25%, 21% had 26–50%, 12% had 51–75% and 3% had > 75% parenchymal involvement [33].

**Table 2:** Demographic characteristics, scanner dose and apnea duration in FDCT and LDCT groups

	FDCT	LDCT	*P* value
Total examinations	269	194	
Period	April–Dec 2020	Dec 2020–March 2021	
*Age*			*P* = 0.8729^a^
Median (IQR)	55 (30)	56 (20)	
[min–max]	[14–92]	[20–96]	
*Sex*			*P* = 0.0357^b^
Male	126 (46.8%)	110 (56.7%)	
Female	143 (53.2%)	84 (43.3%)	
*Weight*			*P* = 0.2501^a^
Mean (SD)	85 (15)	79 (14)	
[min–max]	[60–112]	[59–110]	
DLP (mGy*cm)			*P* < 0.0001^a^
Median (IQR)	341.1 [204.1]	30.8 (3.9)	
[min–max]	[72.6–975.6]	[8.4–71.1]	
*CTDI*			*P* < 0.0001^a^
Median (IQR)	11.3 (5.5)	0.9 (0)	
[min–max]	[0.4–29.8]	[0.3–2.6]	
*Duration of apnea*			*P* < 0.0001^a^
Median (IQR)	13.29 (4.9)	8.27 (1.2)	
[min–max]	[7.02–37.26]	[6.71–15.78]	

DLP, Dose length product; CTDI, CT dose index

^a^Two-tailed Mann–Whitney *U* test

^b^Fisher Exact test

### DLP and duration of apnea

The median DLP was higher 341.1 mGy*cm [IQR, 239.1–443.2] in the FDCT group compared to the LDCT group 30.8 mGy*cm [IQR, 28.9–32.7]. The difference was statistically significant (*P* < 0.0001).

The median duration of apnea was 13.29 s [IQR, 10.85–15.73] for the FDCT group and 8.27 s [IQR, 7.69–8.85] for the LDCT group. The difference was statistically significant (*P* < 0.0001).

### Accuracy of CT compared to biological tests: RT-PCR and rapid antigen test

In the FDCT group, sensitivity for CT to detect COVID-19 was 94% when compared to biological tests, with a specificity of 63.2%. In the LDCT group, sensitivity for CT to detect COVID-19 was 91.2% with a specificity of 36.8%. A separate subgroup analysis for patients who underwent either rt-PCR or rapid antigen testing is represented in Table 3.

**Table 3:** Diagnostic performance of CT Chest compared to biological testing (BT) of COVID-19

	*N* =	CT + BT +	CT-BT-	CT + BT-	CT-BT +	Se	Sp	PPV	NPV	Accuracy (%)
*RT-PCT or Ag test*										
FDCT	69	47	12	7	3	94%	63.2%	87%	80%	85.5
LDCT	99	73	7	12	7	91.2%	36.8%	85.9%	50%	80.8
*RT-PCR*										
FDCT	27	14	9	3	1	93.3%	75%	82.3%	90%	85.2
LDCT	27	22	0	2	3	88%	NC	91.7%	NC	81.5
*Ag test*										
FDCT	42	33	4	3	2	94.3%	57.1%	91.7%	66.7%	88.1
LDCT	72	51	7	10	4	92.7%	41.1%	83.6%	63.6%	80.6

BT, Biological test; FDCT, full dose CT scan; LDCT, ultra-low dose CT scan; RT-PCR, reverse transcriptase polymerase chain reaction; Ag, Antigen; Se, Sensitivity; Sp, Specificity; PPV, Positive predictive value; NPV, Negative predictive value; NC, Not calculated

## Discussion

The current study demonstrated that performing an ultra-low dose CT chest for COVID-19 patients on a 16-row multidetector CT scan was feasible, enabled a 90% reduction in estimated dose and 38% in apnea duration. The average DLP in our study was higher than the results described by Kang et al. [7] (14.5 mGy-cm) and Agostini et al. [19] (19.5 mGy-cm). However, they were much lower than the dose of a standard chest CT-scan protocol DLP (129.1 mGy-cm) and effective dose (1.81 mSv) [6]. Furthermore, our numbers were in the same range of the values of 1 mSv reported by Homayounieh et al. in a 54 medical institutions survey for COVID-19 CT-scans [20].

We found that both LDCT and FDCT are highly sensitive tests for the detection of COVID-19 infection, however the specificity varied significantly being moderate for FDCT and poor for LDCT. Our findings are consistent with the data reported previously by Fang et al. [12], but contrast with a subsequent study by Dagnis et al. that showed LDCT to have a much higher specificity [10]. The discrepancy may be related to the difference in accuracy of the biological tests used. A high false negative rate of CT in the early phase of the disease has been demonstrated [8, 34]. On the other hand, the sensitivity could be overestimated and the specificity compromised because of the high pre-test likelihood of having COVID-19 during the pandemic [35]. Furthermore, the biological tests represent an imperfect reference standard with reports of sensitivity around 70% for rt-PCR [12, 16, 36, 37]. This suggests that the false positive rate of Chest CT may be lower than reported. However, a consensus standard of reference including both techniques has not been performed.

This study has several limitations. First, we acknowledge the inherit selection bias of a retrospective study. Second, our study was performed in a single institution with limited resources, having a 16-row multidetector CT. More resourceful institutions that use higher generation CT scanners with very short acquisition times, newer detector technology with iterative reconstructions or AI-based algorithms may obtain further significant reduction of the radiation dose in a very short time [7, 19, 38, 39]. Third, the CT reading was performed by one of the two radiologists who were not always blinded to the biological study result and interobserver variability was not assessed. Fourth, the standard of reference is heterogeneous including antigen testing and rt-PCR, as the latter was not always available. As mentioned earlier both tests are imperfect reference standard [11, 12, 16, 36]. Fifth, the study design did not enable an assessment of diagnostic performance of LDCT in comparison to FDCT, which necessitates a simultaneous acquisition of both techniques for all the patients. However, in this study, the sensitivity of LDCT was comparable to previous studies [10, 12]. Lastly, no correlation was performed between CT findings and the clinical severity or patient’s outcome.

## Conclusion

In conclusion, our data show that the implementation of a LDCT protocol on a 16-row MDCT achieved a 90% reduction of estimated dose and a 38% reduction in apnea duration without compromising diagnostic accuracy. This has a potential impact on patient triage and management, especially young patients and for those who need repetitive follow-up. Further studies are needed to address the false negative rate of LDCT compared to FDCT.

## Data Availability

The data supporting the findings of this study are available within the article and its supplementary materials as well as in the internal PACS of the hospital.

## Abbreviations

FDCT: Full dose CT
LDCT: Ultra-low dose CT
DLP: Dose length product
IQR: Interquartile range
WHO: World Health Organization
RT-PCR: Reverse transcriptase polymerase chain reaction
ALARA: As low as reasonably achievable
KV: Kilovolts
mAs: Milliampere-seconds
CTDI_vol_: CT dose index
